# Use of barbed suture without fashioning the “classical” Wirsung-jejunostomy in a modified end-to-side robotic pancreatojejunostomy

**DOI:** 10.1007/s00464-020-07991-w

**Published:** 2020-10-06

**Authors:** Luca Morelli, Niccolò Furbetta, Desirée Gianardi, Simone Guadagni, Gregorio Di Franco, Matteo Bianchini, Matteo Palmeri, Caterina Masoni, Giulio Di Candio, Alfred Cuschieri

**Affiliations:** 1 General Surgery Unit, Department of Surgery, Translational Research and New Technologies in Medicine, University of Pisa, Via Paradisa 2, 56124 Pisa, Italy; 2 EndoCAS (Center for Computer Assisted Surgery), University of Pisa, Pisa, Italy; 3 Vascular Surgery Unit, Department of Cardiovascular Surgery, University of Pisa, Pisa, Italy; 4 Institute for Medical Science and Technology, University of Dundee, Dundee, Scotland, UK

**Keywords:** Robotic surgery, Pancreatic surgery, Pancreatojejunostomy, Barbed suture, Pancreatoduodenectomy, Video report

## Abstract

**Background:**

The treatment of the pancreatic stump is a critical step of pancreatoduodenectomy (PD). Robot-assisted surgery (RAS) can facilitate minimally invasive challenging abdominal procedures, including pancreatojejunostomy. However, one of the major limitations of RAS stems from its lack of tactile feedback that can lead to pancreatic parenchyma laceration during knot tying or during traction on the suture. Moreover, a Wirsung-jejunostomy is not always easy to execute, especially in cases with small diameter duct. Herein, we describe and video-report the technical details of a robotic modified end-to-side invaginated robotic pancreatojejunostomy (RmPJ) with the use of barbed suture instead of the “classical” Wirsung-jejunostomy.

**Methods:**

The RmPJ technique consists of a double layer of absorbable monofilament running barbed suture (3–0 V-Loc), the outer layer is used to invaginate the pancreatic stump. Thereafter, a small enterotomy is made in the jejunum exactly opposite to the location of the pancreatic duct for stent insertion (usually 5 Fr) inside the duct. The internal layer provides a second barbed running suture placed between the pancreatic capsule/parenchyma and the jejunal seromuscular layer.

**Results:**

A total of 14 patients underwent robotic PD with RmPJ at our Institution. The mean console time was (281.36 ± 31.50 min), while the mean operative time for fashioning the RmPJ was 37.31 ± 7.80 min. Ten out of 14 patients were discharged within postoperative day 8. No clinically relevant pancreatic fistulas were encountered, while two patients developed biochemical leaks.

**Conclusions:**

RmPJ is feasible and reproducible irrespective of pancreatic duct size and parenchyma, and can enhance the surgical workflow of this operation. Specifically, the use of barbed sutures allows the exploitation of the potential advantages of the RAS, while minimizing the negative effect caused by the main disadvantage of the robotic approach, its absence of tactile feedback, by ensuring uniform tension on the continuous suture lines used, especially during the reconstructive phase of the operation.

**Electronic supplementary material:**

The online version of this article (10.1007/s00464-020-07991-w) contains supplementary material, which is available to authorized users.

Pancreatoduodenectomy (PD) is a complex surgical procedure, the execution of which is nowadays standardized. The resective part of the procedure remains broadly similar to that originally described by Whipple [1], with some modifications reported in different series. In contrast, the reconstructive part, and particularly the management of the pancreatic stump, still lacks universal standardization [2–4]. In this respect, the failure of pancreatic anastomosis remains a problem as it inevitably leads to the development of postoperative pancreatic fistula (POPF), the most feared complication. To date, no consensus has been reached on the optimal technique for execution of this anastomosis, which remains the Achilles heel of PD.

This issue concerns both open and minimally invasive surgery as the surgeon must consider the technical difficulties specifically related to the technique used for dealing with the pancreatic stump. In this respect, different anastomosis techniques have been proposed for laparoscopic pancreatojejunostomy (PJ) [5, 6]. Robotic assisted surgery (RAS) has been considered an evolution of pure laparoscopy, capable of overcoming some of its intrinsic limitations of direct manual laparoscopy. The advantages provided by RAS include provision of seven degrees of freedom of the instruments, the stable, immersive, stereoscopic high-definition imaging of the operating field, restoration of the eye-hand coordination, and improved surgical dexterity. All these advantages have led to a progressive increased uptake of in robotic pancreatic surgery.

Several techniques of pancreatic anastomosis have been proposed, including pancreato-gastrostomy (PG) and different techniques of PJ, including duct to mucosa and invagination [7–12]. Undoubtedly, one of the limitations of RAS for PD relates to the lack of a tactile feedback, since this may contribute to iatrogenic pancreatic parenchymal laceration during knots tying or from suboptimal suture line tension during continuous suturing [13]. Furthermore, a Wirsung-jejunostomy is not always easy to execute, especially in the presence of very small diameter pancreatic duct.

In this report, we describe and video-report the technical details of a Robotic modified end-to-side, invaginated PancreatoJejunostomy (RmPJ) with the use of continuous barbed sutures. This technique replaces the “classical” Wirsung-jejunostomy.

## Methods

The RmPJ technique represents a variant of our previously described technique [14], specifically modified for RAS, and consists of a double layer of monofilament absorbable running barbed suture (3–0 absorbable V-Loc Wound Closure Device, Medtronic), with stenting of the small jejunal enterotomy and the pancreatic duct.

### Informed consent and review board approval

All patients provided informed consent for surgery and for the anonymous use of videos and photographs of the procedures, for scientific or training purposes.

The study was approved by the Institutional Review Board.

### Docking and instruments

The patient is placed supine with the legs parted. The assistant surgeon stands between the patient’s legs. A total of five trocars are placed at least 8 cm aside from each other to minimize the risk of collision of manipulator arms. Four robotic trocars are placed about 1–2 cm above the transverse umbilical line, two along the mid-clavicular line and two on the anterior axillary line, on either side. The 12-mm assistant port is placed immediately below or above the umbilicus, depending on the distance between the xiphoid process and the umbilicus. A right mid-clavicular line trocar is used for the camera. The robot is docked from the right side of the patient.

Monopolar scissors, bipolar Maryl and forceps, and a grasper are used routinely for the right hand, the left hand, and the fourth arm, respectively. In addition, the EndoWrist Vessel Sealer Extend is used for the dissection. One needle driver only is used in the right hand for the anastomoses. The dissection phase of the procedure follows the steps of open surgery, with some refinements needed for RAS approach.

### Technical details of RmPJ

The technique is similar in many respects to our previously described modified PJ technique [14] in open surgery, with some modifications and technical refinements needed for the RAS technique.

Video 1. As in open surgery, the transection of the pancreas is made vertically, using monopolar scissors, with careful hemostasis aided with bipolar Maryland forceps. The pancreatic stump is freed by only about 1–2 cm from the splenic artery and vein. The jejunal limb with the largest diameter is obtained by transecting it just distal to the Treitz ligament, approaching it from the right side. The initial step consists in determining the best position for the invagination of the jejunal limb, lying it in front of the freed 1–2 cm pancreatic stump.

**Fig. 1:**
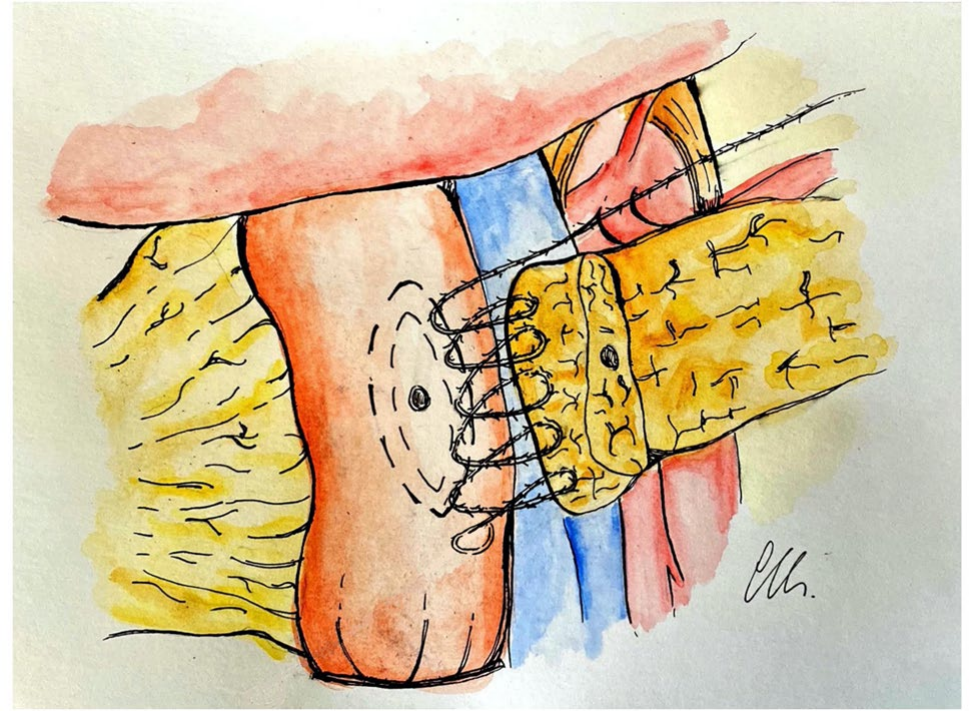
Outer posterior layer

Video 2. The anastomosis begins with a monofilament absorbable running barbed suture (V-Loc 3–0), starting at the postero-inferior surface of the pancreas. The posterior surface of the pancreas is sutured to the seromuscular layer of the jejunum about 10 mm from the transection of the pancreas. The sutures are placed transversally through the pancreas and then the jejunum. The running suture is progressively tightened, after each passage through the jejunum (Fig. 1).

**Fig. 2:**
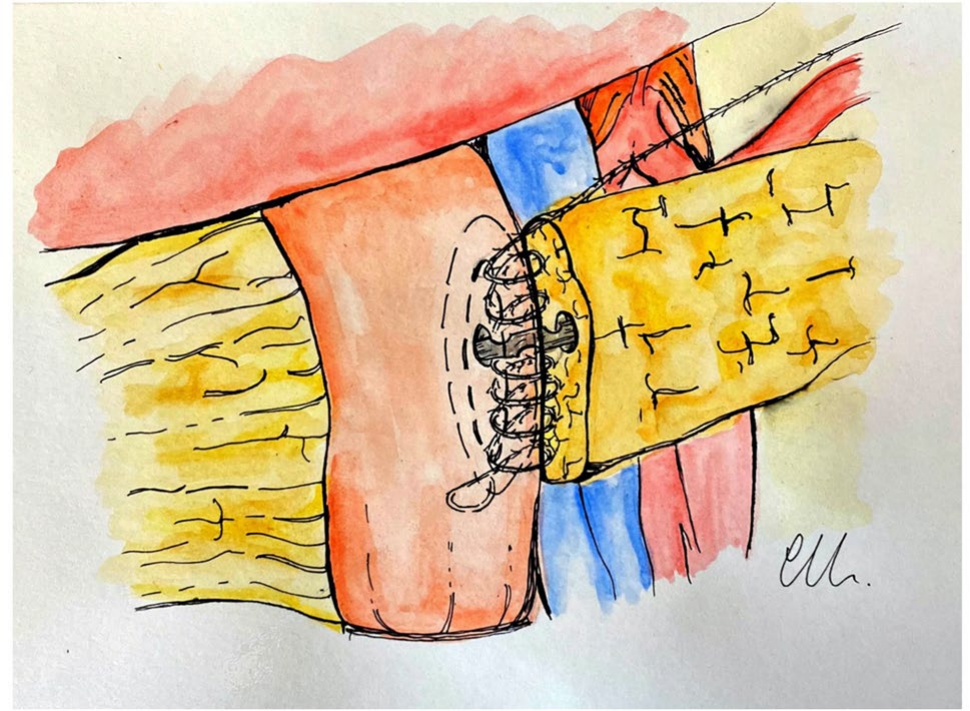
Inner posterior layer

A second posterior layer also using barbed suture is placed between the posterior transected surface of the pancreas (capsule and parenchyma), and the seromuscular layer of the jejunum just above the previous suture. The suture on the posterior wall is progressively tightened and the barbed suture again maintains the tension throughout the suture bites (Fig. 2).

After completing the posterior layer, the monopolar scissors are used to create a small enterotomy in the jejunum of equivalent size to the pancreatic duct, after ensuring that the location of the enterotomy lies exactly opposite to the pancreatic duct. Then, a ureteral stent (usually 5 French) is placed with the straight end in the Wirsung duct and the pigtail end in the jejunal lumen.

**Fig. 3:**
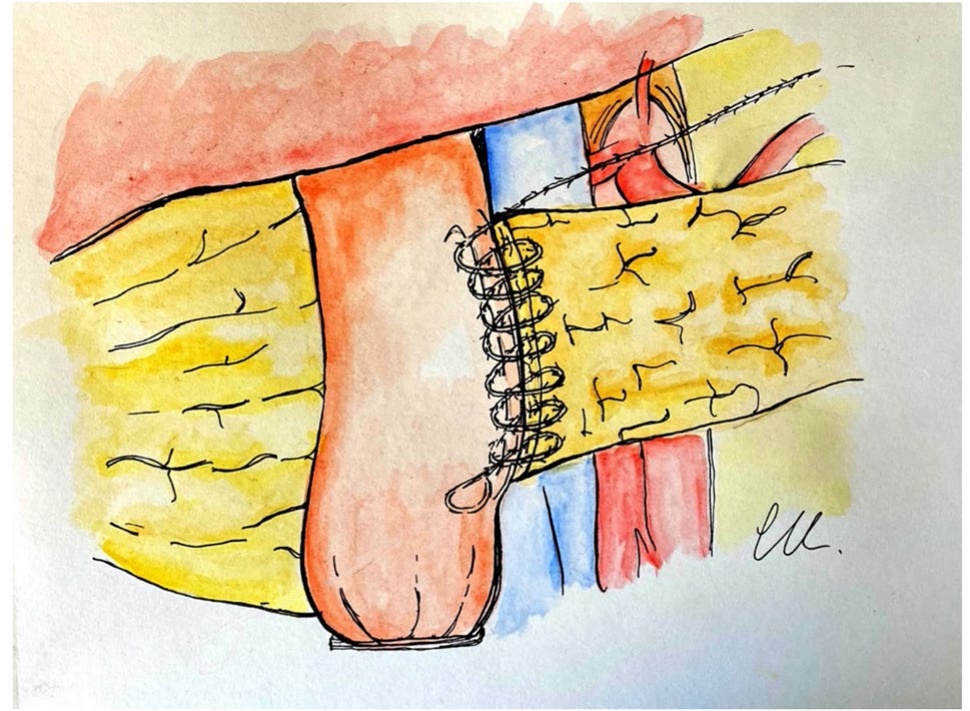
Inner anterior layer

Next, the inner anterior layer is performed with a continuous running suture between the anterior pancreatic transected surface (capsule and parenchyma) and the anterior seromuscular layer of the jejunum. Again, the suture is tightened step by step, after each passage through the jejunum. When the halfway point is reached, we prefer to start with a new running suture from the upper apex of the anastomosis. This is needed because the corners are the most delicate points and progressive tightening the suture would hinder the view of the upper corner. The elliptical line of the inner layer is completed in this way (Fig. 3).

**Fig. 4:**
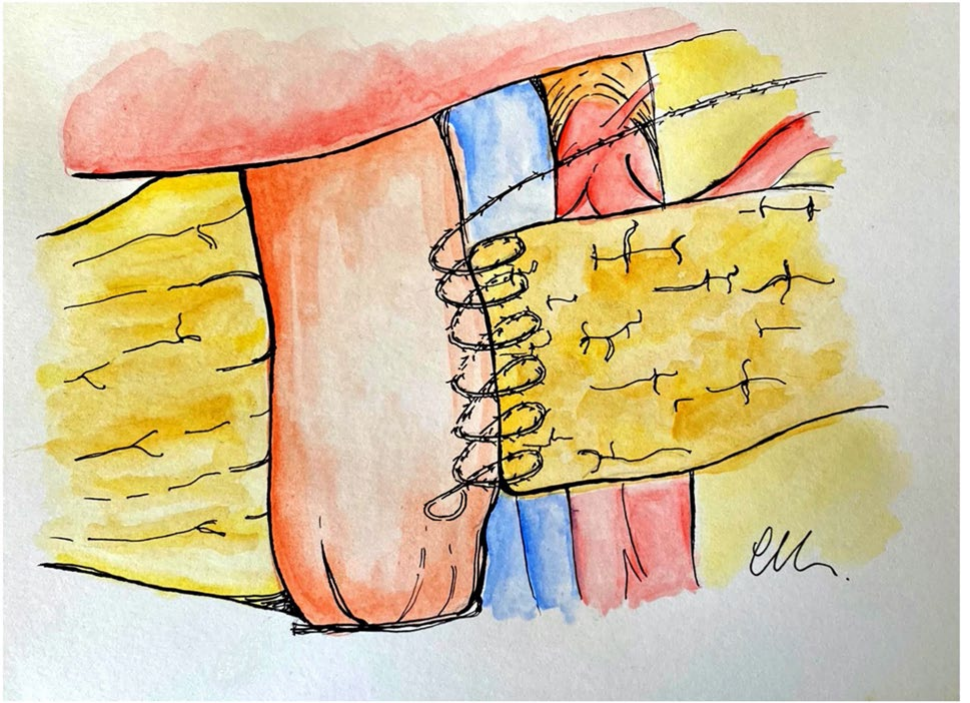
Outer anterior layer

**Fig. 5:**
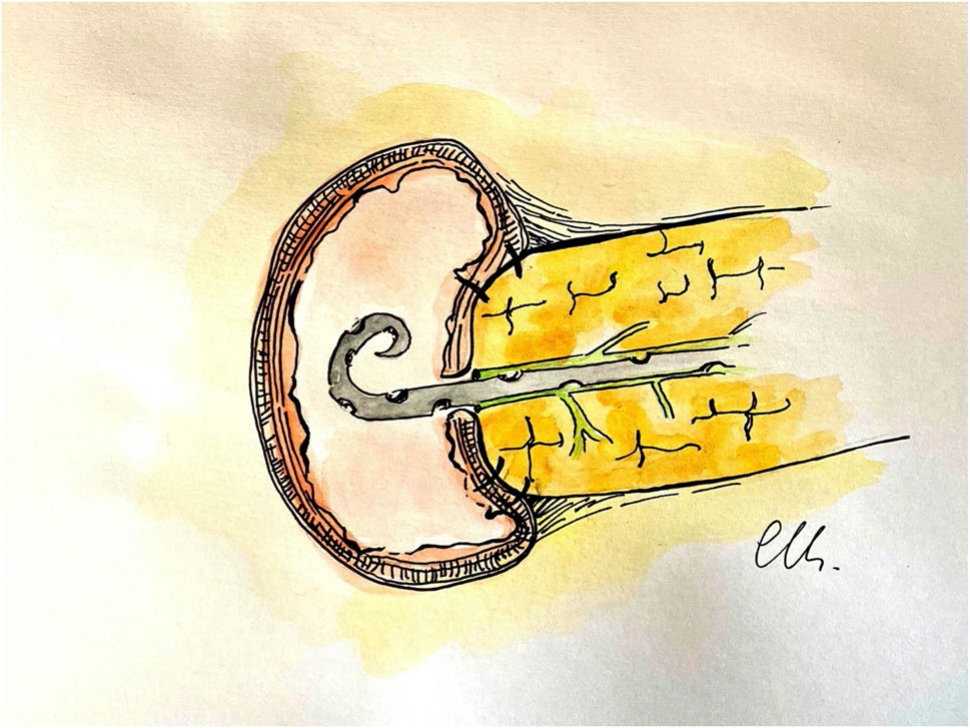
Final view of the completed RmPJ anastomosis

Finally, the anastomosis is concluded with the external anterior invaginating layer, with the last row of running barbed suture. Each bite starts with the needle entry in the anterior pancreas (capsule and parenchyma) approximately 10 mm from the anterior transected surface. The needle should exit from the pancreas 5 mm away from the initial entry point into the organ. The suture is completed with the second passage through the jejunum. In this way, as the sutures are pulled with adequate tension and tightened, the jejunum rolls over onto the pancreas. These sutures are placed approximately 3–5 mm apart such that they imbricate the anterior seromuscular jejunum over the pancreas (Figs. 4, 5).

### Postoperative management

The nasogastric tube is removed, and patients are usually extubated at the end of the procedure. Prophylactic somatostatin or somatostatin analogs are not administrated routinely in the postoperative period.

Evaluation of POPF, management of peritoneal drains, and treatment of fluid collection are standardized and in accordance with latest guidelines.

The drain output volume is measured daily, and its amylase content is assayed on postoperative day (POD) 3 and 5, and when positive, every 3 days until drain removal.

POPF is diagnosed and classified according to the 2016-ISGPS criteria [15]. The drainage tubes are removed on POD 5 in patients judged as ISGPS grade none, negative amylase content of the drainage and without any signs of intra-abdominal infection. Abdominal ultrasound exam is performed as first level exam in cases of clinical suspicion of intra-abdominal complications and followed by computed tomography when indicated. Intra-abdominal collections caused by POPF are drained with an interventional ultrasound-guided procedure, usually with the placement of a pigtail catheter in the first instance, with CT-guided pigtail placement being reserved for failed ultrasound-guided procedure. Amylase activity is also measured in fluid samples obtained by aspiration of intra-abdominal collections or ascites.

## Results

Against a cohort of more than 200 PJ performed with the open approach of the technique, a total of 14 patients underwent robotic PD with RmPJ. The group consisted of 8 females and 6 males; mean age was 67.12 ± 6.88 years and mean BMI was 23.91 ± 4.09 kg/m^2^. One patient had ASA (American Society of Anesthesiologists) score of 1, 3 had ASA score of 2, and 10 patients had ASA score of 3. The mean console time was (281.36 ± 31.50 min), while the mean operative time for fashioning the RmPJ was 37.31 ± 7.80 min. Nine patients had soft pancreas and 10 had Wirsung duct ≤ 2 mm. Five patients were diagnosed with pancreatic adenocarcinoma (PDAC), while 3 were diagnosed with periampullary adenocarcinoma, 4 with intraductal papillary mucinous neoplasm (IPMN), and 2 with duodenal adenocarcinoma. The mean Fistula Risk Score (FRS) [16, 17] was 4.71 ± 1.81. The average length of hospitalization was 9.28 ± 3.60 days; 10 out of 14 patients were discharged within POD 8. No clinically relevant POPFs were encountered, while two patients developed biochemical leaks (BL, 2/14, 14.3%), both with positive amylase content only on POD 3, and drainage removal on POD 5 with negative amylase levels. Four patients had delayed gastric emptying (DGE) requiring re-insertion of the nasogastric tube on POD 2 with its removal on POD 10, 15, 13, and 14, respectively. One patient experienced an episode of transient hyperamylasemia, which resolved spontaneously.

## Discussion

Although the enabling features of RAS technology have facilitated uptake of the minimally invasive approach even for pancreatic surgery, its lack of haptic feedback constitutes the Achilles heel of this versatile master-salve technology, because it may lead surgeons to place excessive tension while tying sutures, iatrogenic tearing of the tissues being sutured, or rupture of the suture itself [13]. This limitation is particularly evident in pancreatic surgery where tight knots on the parenchyma can tear the pancreas, causing postoperative leaks with development of POPF.

Possibly, visual feedback may help to some extent in overcoming the loss tactile feedback, certainly for expert surgeons [18], but it still remains the issue of the current generation of robotic technologies.

The technique described in 2017 by our group [14] for the open surgery has been proven to be safe and has resulted in a lower POPF rate than expected, especially for “difficult” pancreas with high FRS, including soft gland texture and narrow pancreatic duct. Since the publication, we have performed more than other 100 open PD with the same good results. Against this experience, we developed a robotic variant by reproducing the main features of the reported open technique; essentially by tailoring the open technique to the robotic platform, to exploit the advantages of the robotic technology. The advantages of RAS, including the instruments articulation degrees and the 3D visualization, are used to reproduce the double layer performed by conventional open surgery. The lack of tactile feedback has been overcome to some extent with the use of barbed suture.

The main key points of the RmPJ are as follows:

1. The jejunum is sectioned very near to the Treitz ligament, the widest segment of the jejunum.
2. The mobilization of the pancreatic stump is limited to 1–2 cm from the splenic artery and vein.
3. The 3/0 barbed suture is used for all the 4 layers of the anastomosis.
4. Running sutures are tightened progressively, after each passage through the jejunum.
5. The small enterotomy is placed exactly opposite to the location of the pancreatic duct, without placing any sutures in the pancreatic ductal epithelium, but instead opposing and stenting them.

The use of the widest segment of the jejunum ensures an easier termino-lateral invagination of the pancreatic stump. Indeed, this is an important prerogative for a successful invagination technique, as it allows the jejunum to envelop the pancreatic stump and to roll over onto the pancreas. Obviously, the extent of the invagination varies, depending on the individual size variability of the jejunum, but in our experience, with the described technique, it is generally sufficient to obtain the desired result.

The limited mobilization of the pancreas avoids the devascularization of the pancreatic stump, leaving the minimum necessary space on posterior surface necessary for the desired invagination.

The use of barbed sutures has proven to be safe and timesaving in general surgery and urology [19–21], but its use has not yet been reported in recent systematic reviews of PJ techniques [10–12].

So far, this application has been described only in few cases of minimally invasive pancreatic surgery. Indeed, while only two publications have reported the use of barbed sutures for laparoscopic and robotic lateral PJ performed to treat chronic pancreatitis [22, 23] and another two for laparoscopic PJ after PD [24, 25], a barbed suture has never been used to perform robotic PJ during PD.

In RmPJ, barbed sutures are used for all the 4 layers of the anastomosis, specifically to overcome the lack of tactile feedback during tightening of the suture. Indeed, as the characteristics of the barbed suture allows maintenance of the required suture tension after each passage, we think that this aspect may play a key role in enhancing the safe execution of RAS pancreatic surgery.

In practice, the running sutures are tightened progressively, after each passage through the jejunum, ensuring that the sutures are exposed to a uniform optimal tension when tightened, which reduces the risk of pancreatic parenchyma and capsule laceration.

Some surgeons have raised doubts regarding the use of these sutures for fragile tissues, because of their thickness, and the theoretically more traumatic needle. These considerations may be of particular concern in cases with soft pancreas, from possible increased risk of tissue laceration and leakages of pancreatic juice. Although at the beginning of our experience we shared these concerns, we have progressively acquired ease and confidence with barbed sutures by using them on 9 soft pancreases. We have not experienced any laceration, and thanks to the tightening of the suture, the jejunum looked like being glued to the pancreas, giving a visual appearance of particular strength to the anastomosis. The result of the absence of any intraoperative parenchymal damage is in line with the absence of clinically relevant fistula in the postoperative course.

Two other key points supporting the use of barbed suture in RmPJ are: (i) these sutures have the required tensile strength, which is rarely weakened by continued traction or grip with robotic instruments, as distinct from other monofilament sutures, e.g., Prolene, which are more prone to rupture and (ii) as running sutures, they are timesaving and, therefore, can enhance the surgical workflow, compared to the interrupted sutures used in the majority of the anastomotic techniques.

We think that all these characteristics should be taken into account when robotic assistance is used in performing complex operations such as a PD particularly for the reconstructive phase of the operation.

In performing laparoscopic PJ after PD, De Pastena et al. [24] described the use of 4 barbed sutures passing through the pancreas, according to the Blumgart technique, while Edil et al. [25] reported excellent results with the use of barbed sutures in performing running single-layered anastomosis, with a pediatric feeding tube placed from the pancreatic duct into a small enterotomy approximated to the pancreatic duct. Although the latter laparoscopic technique has some similarities to our technique, the two important differences are the double layer with an external invagination vs. the single layer, and the use of the robotic assisted vs. the laparoscopic approach.

Interestingly, a recent paper describing a reduction of clinically significant pancreatic leaks with the use of barbed sutures in performing open PJ after PD [26] is perfectly in line with the excellent ones reported by Edil et al. [25], and with those that we have registered with RmPJ, enforcing our suggestion to extend the use of barbed sutures to the robotic technique, since this may represent an important further specific advantage.

As reported in the open technique [14], the small incision in the jejunum without suturing the Wirsung duct to the jejunum, which is stented instead, are two hallmarks of the RmPJ that, in our opinion, combines the advantages of the duct-to-mucosa and invagination techniques used in open surgery. As distinct from the stent used in the duct-to-mucosa technique, the stent function in RmPJ is to maintain alignment of the Wirsung duct with the small incision in the jejunal wall.

Although RAS compared to open surgery is generally considered to facilitate execution of duct-to-mucosa anastomosis, the recognized limitation of duct-to-mucosa PJ, the issue arises in cases with a small diameter pancreatic duct, with increased risk of obstruction or fistula. The technique reported obviates this problem by avoiding the creation of a “classical” Wirsung-jejunostomy.

A possible concern in this regard, could be the consequences if stent dislocation or delayed retention occurs. However, as several studies have not reported these specific clinical issues after PJ [11, 27], and so far, we have not encountered any stent-related clinically relevant issues, it seems unlikely that this could materially influence clinical outcomes. In any case, this specific aspect of RmPJ needs to be evaluated on larger series, and with long-term follow-up, to enable robust conclusions.

The main limitations of our study are the cohort size, the possible bias selection, the retrospective nature, and the lack of a control group.

Indeed, in the present series of patients who underwent RAS, we selected cases without preoperative signs of vascular infiltration, without previous major abdominal surgery, and no contraindications to pneumoperitoneum. This may have influenced the postoperative outcome in terms of low postoperative morbidity. However, the smaller tumors or those of the ampullary region are related to a higher FRS (soft gland, thin Wirsung duct, and non-adenocarcinoma diagnosis) and this selection, although it can result in an easier resective phase of the operation, it is unlikely that could have introduced bias in favor of the PJ technique. Although the zero rate of clinically relevant fistulas documented in these patients, must be viewed with caution because of the small cohort size, it still represents an encouraging outcome in our opinion, particularly if we consider the FRS of our patients, and the results currently reported in the available systematic reviews on robotic PD [12, 28]. We accept fully that the favorable outcome reported in this initial small series of RmPJ need confirmation by a larger prospective series.

## Conclusions

In our opinion, RmPJ is feasible and reproducible irrespective of pancreatic duct size and parenchyma and can enhance the surgical workflow of this operation. Specifically, the use of barbed sutures allows the exploitation of the potential advantages of the RAS, while minimizing the negative effect caused by the main disadvantage of the robotic approach, its absence of tactile feedback, by ensuring uniform tension on the continuous suture lines used especially during the reconstructive phase of the operation.

## Electronic supplementary material

Below is the link to the electronic supplementary material.

Supplementary file1 (MP4 326896 kb)

Supplementary file2 (MP4 504322 kb)
